# New records of the land and freshwater molluscs of Gran Canaria (Canary Islands, Spain)

**DOI:** 10.3897/zookeys.985.53974

**Published:** 2020-11-05

**Authors:** Ward Langeraert, Dimitri Brosens

**Affiliations:** 1 Ghent University (UGent), Ghent, Belgium Ghent University Ghent Belgium; 2 Research Institute for Nature and Forest (INBO), Brussels, Belgium Research Institute for Nature and Forest Brussels Belgium; 3 Belgian Biodiversity Platform, Brussels, Belgium Belgian Biodiversity Platform Brussels Belgium

**Keywords:** freshwater, Gran Canaria, observation, occurrence, open data, snails, terrestrial

## Abstract

“Land and freshwater molluscs of Gran Canaria (Spain)” is an occurrence dataset containing 389 observations of 59 different taxa of land and freshwater molluscs encountered on Gran Canaria, an island central in the Canarian archipelago (Spain). Of these 59 different (sub)species, 27 are with certainty currently endemic to the island of Gran Canaria. Various sites were inspected in a period between 1988 and 2020. The dataset is published as a standardized Darwin Core Archive and includes for each observation a stable occurrence ID, scientific name, date, and location of the observation, as well as information on life stage and organism quantity. It also contains supplementary remarks on the determination and the observation itself and links to associated media. We have released this dataset to the public domain under a CC0 1.0 Universal (CC0 1.0) Public Domain Dedication. The aim is to contribute to the knowledge on the ecology and distribution of these species on the island, such that it may aid conservation and research of these organisms in the future.

Issues with the dataset can be reported at https://github.com/BelgianBiodiversityPlatform/landsnails-occurrences

## Rationale

**Figure 1.**
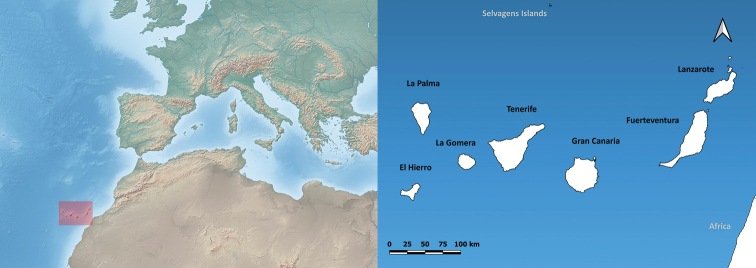
Map showing the Canary Islands (Spain) situated in the Western Palearctic region. Gran Canaria is located in the centre of the archipelago.

Canary Islands (Spain) is an archipelago off the coast of northwest Africa. Gran Canaria is the third-largest island and located in the centre of the archipelago (Carracedo and Troll 2016; Fig. 1). The island contains a number of endemic land snail species (e.g. Brito and Fraga 2010). Freshwater molluscs are of lesser significance, but are also present. Research on land and freshwater molluscs of the Canaries mainly goes back to the 19^th^ century with the famous works of Webb and Berthelot (1833), Shuttleworth (1852a, 1852b), Mousson (1872), Wollaston (1878), Mabille (1884), Odhner (1931), and others. Recent checklists are available (Groh 1985; Bank et al. 2002; Brito and Fraga 2010; Helixebas 2019) and some more recent papers are cited further in this article. Nevertheless, taxonomic research is still largely based on these old works and many species have never been found again since their description, or the ecology or proper range is not known. This, together with the threats of global warming (Luque et al. 2014) and the increase of demographic and touristic pressure (Ibáñez et al. 1997), could have (and probably already has) detrimental consequences for the survival of these species on the island (see also the assessments of IUCN Red List of Threatened Species (https://www.iucnredlist.org/)). Therefore, there is an urgent need for information on ecology, distribution and taxonomy. We hope that this dataset of land and freshwater snail occurrences can contribute to the knowledge on these species and ensure their survival on Gran Canaria and the Canary Islands as a whole.

## Taxonomic coverage

**Figure 2.**
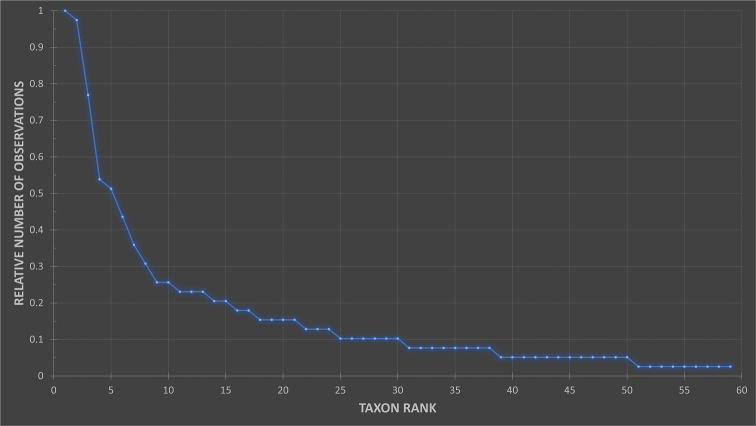
Graph showing the relative number of observations for each taxon. Taxon rank: 1 = most observed (sub)species, 2 = second most observed (sub)species etc.

**Figure 3.**
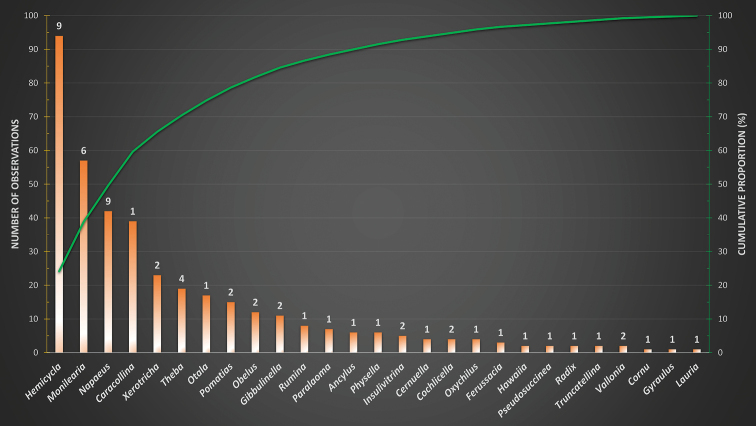
Graph showing the number of observations per genus and the cumulative proportion of observations. Numbers above the bars indicate the number of different taxa observed per genus.

Land and freshwater molluscs do not comprise a monophyletic taxonomic group, but are all mollusc species that live in respectively land and freshwater habitats. On land, only gastropods occur (class Gastropoda, snails and slugs) and in freshwater, both Gastropoda and bivalves (class Bivalvia) occur. No observations on bivalves are present in this dataset. The dataset includes 389 observations of 59 species and subspecies (Fig. 2) belonging to 27 genera (Fig. 3) and 18 families. Of these 59 taxa, 37 are with certainty currently endemic to the Canary Islands whereas 27 are endemic to the island of Gran Canaria in particular and 3 also occur on other islands. It is unclear whether the remaining 7 endemic taxa found on Gran Canaria also occur on other islands or not.

Species determination was mainly done during two meetings concerning the land snails of Gran Canaria organized by the Dutch Malacological Society (Nederlandse Malacologische Vereniging, NMV), coordinated by Theo Ripken. These meetings took place on 21 April 2018 and 23 February 2019 in, respectively, Leiden and Den Haag (Netherlands). Reference material and expert knowledge provided many identifications. Determinations were further based on the following articles: Alonso et al. (1995), Alonso and Ibáñez (2015b), and Yanes et al. (2011) for the genus *Napaeus*, Alonso and Ibáñez (2015a) and Valido et al. (1990) for *Insulivitrina*, Ibáñez et al. (2003) for the genus *Obelus*, Gittenberger and Ripken (1987) for the genus *Theba*, and Hutterer and Groh (1991) for the genus *Truncatellina* (see also Langeraert 2019). Also, Mousson (1873) (first published in 1872, see Bank et al. 2002), Wollaston (1878), Mabille (1884), Odhner (1931), Shuttleworth (1975), Groh et al. (1992), Neubert and Gosteli (2003), Serna and Gómez (2008), and Neiber (2015) were consulted. Additional information was found in Groh (1985), Bank et al. (2002), Brito and Fraga (2010), Helixebas (2019) and as well on https://www.malacowiki.org/ and https://www.iucnredlist.org/. Finally, for species with a wider (European) distribution, the following works were consulted: Cameron (2008), Welter-Schultes (2012), Horsák et al. (2013), Glöer (2015), Jansen (2015), and Cadevall and Orozco (2016).

## Taxonomic ranks

Taxonomy is according to MolluscaBase eds. (2020) except for the taxon *Pomatias adjunctus* (Mousson, 1872). This species is known under the name *Pomatias canariensis* (d’Orbigny, 1840) in that database, but this name is a synonym of *Pomatias laevigatus* (Webb & Berthelot, 1833), which is a species other than *P. adjunctus* (see below; Theo Ripken personal comment; Yanes et al. 2004).

**Kingdom**: Animalia

**Phylum**: Mollusca

**Class**: Gastropoda

**Families**: Achatinidae, Enidae, Ferussaciidae, Geomitridae, Helicidae, Lauriidae, Lymnaeidae, Oxychilidae, Physidae, Planorbidae, Pomatiidae, Pristilomatidae, Punctidae, Streptaxidae, Trissexodontidae, Valloniidae, Vertiginidae, Vitrinidae

**Species**: *Ancylus striatus*, *Caracollina lenticula*, *Cernuella virgata*, *Cochlicella acuta*, *Cochlicella barbara*, *Cornu aspersum*, *Ferussacia folliculum*, Gibbulinella aff. dealbata, Gibbulinella aff. dewinteri, *Gyraulus parvus*, *Hawaiia minuscula*, *Hemicycla berkeleii*, *Hemicycla ethelema*, Hemicycla cf. gaudryi, *Hemicycla glasiana*, *Hemicycla guamartemes*, *Hemicycla psathyra psathyra*, *Hemicycla psathyra temperata*, Hemicycla psathyra cf. temperata, *Hemicycla saponacea*, *Hemicycla saulcyi carta*, *Hemicycla* spec., *Insulivitrina nogalesi*, *Insulivitrina parryi*, *Lauria cylindracea*, *Monilearia arguineguinensis*, *Monilearia montigena*, *Monilearia phalerata*, Monilearia cf. praeposita, *Monilearia pulverulenta*, *Monilearia tumulorum*, *Monilearia* spec., *Napaeus exilis*, *Napaeus interpunctatus*, *Napaeus isletae*, *Napaeus josei*, *Napaeus moquinianus*, *Napaeus myosotis*, *Napaeus obesatus*, *Napaeus validoi*, *Napaeus venegueraensis*, Napaeus cf. venegueraensis, *Obelus despreauxii*, *Obelus pumilio*, *Otala lactea*, *Oxychilus draparnaudi*, *Paralaoma servilis*, *Physella acuta*, *Pomatias adjunctus*, Pomatias aff. laevigatus, *Pseudosuccinea columella*, *Radix auricularia*, *Rumina decollata*, *Theba arinagae*, *Theba geminata*, *Theba grasseti*, *Theba pisana*, *Truncatellina atomus*, *Vallonia costata*, *Vallonia pulchella*, *Xerotricha conspurcata*, Xerotricha aff. orbignii

## Remarks concerning taxonomic status

Two species of *Gibbulinella* were found on the island: Gibbulinella aff. dealbata and G. aff. dewinteri. The shells of Gibbulinella aff. dealbata are wider and more solid than those of G. aff. dewinteri. These identifications were made on the meetings concerning the land snails of Gran Canaria organized by the Dutch Malacological Society where it was hypothesised that the shells found on Gran Canaria differ from those of *G. dealbata* (Webb & Berthelot, 1833) and *G. dewinteri* Bank, Groh & Ripken, 2002 from other islands. Indeed, we could find no published records of *G. dewinteri* from Gran Canaria and although this species was originally described as *Pupa* (=*Gibbulinella*) dealbata var. minor by Mousson (Bank et al. 2002), our shells are not smaller than *G. dealbata*. They are equally large, but they are slenderer. This genus should be revised on the Canary Islands.

*Hemicycla gaudryi* (d’Orbigny, 1839) and *H. ethelema* (J. Mabille, 1882) show strong similarities but the shells of *H. ethelema* have a characteristic granulation. Our specimens from the Jardín Botánico Viera y Clavijo (botanical garden Tafira Alta) are old shells and it is not clear whether the granulations are lacking because the shells belong to *H. gaudryi* or because they are old and withered. Therefore, we used the name H. cf. gaudryi. Also, the shells were found at the overlap/edges of the distribution areas of both species but the distribution of *H. gaudryi* is not well known. Furthermore, at the meetings of Dutch Malacological Society it was stated that the correct name for this taxon should be *Hemicycla themera* (J. Mabille, 1883) (Theo Ripken personal comment). More research is necessary on this species.

The shells designated as Hemicycla psathyra cf. temperata were found at a location outside the known range of *H. psathyra temperata* (Mousson, 1872) (northwest of the island), but we believe these shells can be contained within morphological variation of *H. psathyra temperata*. Hemicycla psathyra cf. temperata was treated together with *H. psathyra temperata* for the generation of Figs 2, 3 and thus not counted as a separate taxon.

A single shell was identified as *Hemicycla* spec. This is a juvenile shell too small for precise determination. It belongs to either *H. glasiana* (Shuttleworth, 1852) or *Hemicycla guamartemes* (Grasset, 1857). This observation was removed for the generation of Figs 2, 3 and thus not counted as a separate taxon.

In Barranco de Guayadeque, shells were found that are very convex, have an obtuse apex, and are larger than *Monilearia phalerata* (Webb & Berthelot, 1833). This description agrees nicely with the original description of *Monilearia praeposita* (Mousson, 1872) and the location agrees with the range given by the IUCN assessment of this species (Groh and Neubert 2011b). On the other hand, the IUCN status is Data Deficient and in the meetings of Dutch Malacological Society there were some doubts on the status of this species. Therefore, we identified these shells as M. cf. praeposita. The fossil shells from Arinaga look like the shells from Barranco de Guayadeque but they are smaller. More research is necessary on this species.

The name *Monilearia* spec. is applied to *Monilearia* specimens where the shell is diamond shaped and has an open umbilicus. It is however not as high as *M. phalerata*. *Monilearia* spec. was observed in the northwest and from the west to the south of the island. Because of its open umbilicus and its range, this species could be *M. caementitia* (Shuttleworth, 1852). However, because we observed this species outside its known range (Groh and Neubert 2011a) and the uncertain differences with *M. persimilis* (Shuttleworth, 1852) and *M. inops* (Mousson, 1872), we identified this species as *Monilearia* spec. Comparison with reference material from museums would be helpful.

Several shells were found similar to *Napaeus venegueraensis* Artiles, Santana & Deniz, 2011 but smaller and with a more pointed top. These were designated as N. cf. venegueraensis and are possibly subadult shells of *N. venegueraensis*. Napaeus cf. venegueraensis was treated together with *N. venegueraensis* for the generation of Figs 2, 3 and thus not counted as a separate taxon.

The identifications of Pomatias aff. laevigatus were made in the meetings organized by the Dutch Malacological Society where it was hypothesised that the shells found on Gran Canaria differ from the *P. laevigatus* known from Tenerife. Pomatias aff. laevigatus is entirely smooth and occurs in the west of Gran Canaria, while *P. adjunctus* is ribbed and occurs in the northwest-northcentral part of the island. More research on this genus is needed in the Canary Islands.

Shells of Xerotricha aff. orbignii were found at several locations in the west of the island which look very much like *X. orbignii* (d’Orbigny, 1836), a species endemic to Tenerife. No endemic *Xerotricha* species are reported from Gran Canaria in recent checklists (Groh 1985; Bank et al. 2002; Brito and Fraga 2010; Helixebas 2019), but recent articles mention the species from Gran Canaria (Hutterer and Gittenberger 1998; Castillo et al. 2008; Wall et al. 2018). In the latter two citations the name X. aff. orbignii is used for these shells from Gran Canaria. Further research should reveal the relationship between *X. orbignii* from Tenerife and X. aff. orbignii from Gran Canaria.

## Geographic coverage

**Figure 4.**
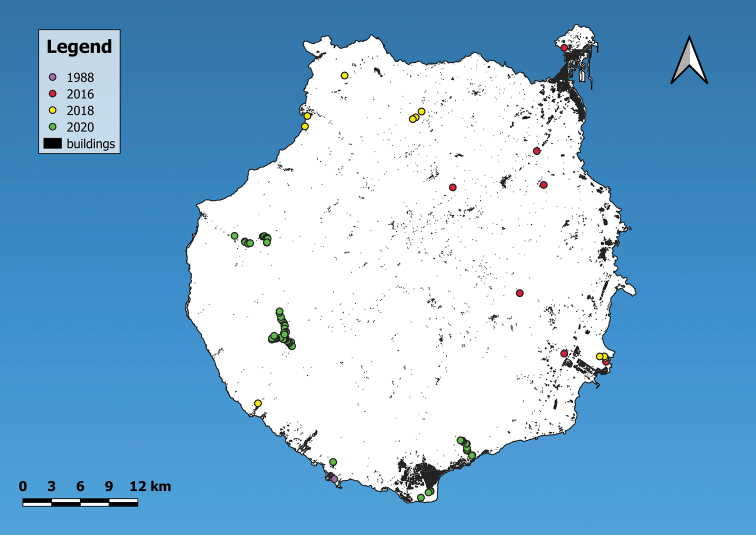
Map of Gran Canaria indicating all different locations in the dataset, coloured according to visiting year.

The dataset comprises three trips taken to Gran Canaria (Canary Islands, Spain; Fig. 4) by the first author in the period between 2016 and 2020 and three shells of *Monilearia arguineguinensis* (Seddon & Aparicio, 1998) collected in 1988, that were obtained as a gift (WL:SNAIL:GC:OCC:00000).

## Bounding box

West: 15°46,9642'W; East: 15°23,3691'W; North: 28°9,6014'N; South: 27°44,3515'N

## Temporal coverage

1988-04-08 to 2020-02-08

## Methodology

### Sampling description

Sampling was done at random along random routes. Locations were not predefined, but some regions were specifically visited because of known species richness or the occurrence of endemics. On site, observations were incidental, but microhabitats or elements that were thought to be favourable for snails were given more attention (e.g. dead wood, north facing slopes etc.). Collection of specimens was mainly done by hand on sight. In some cases, a soil sample was taken that was later examined at home.

Individuals were observed as living snails or empty shells (recent or (sub)fossil). Juveniles were treated as individuals with a shell that lack adult characteristics, like an underdeveloped peristome or the presence of a keel that is not present in adult shells. Following this practice, subadult specimen were often classified as juveniles.

### Method step description

The following steps were used from data collection up to final data publication:

1. Data collection Data sampling as described aboveSpecies name, date, location, organism quantity, and additional remarks were noted in the field in a field notebook or entered in the app ObsMapp (Observation International 2019) (for occurrences in 2020)Later, final determinations were conducted as described earlier and additional information was noted on organism quantity, location, and identification remarksFor shells in collection, data was kept on a label along with each specimenFor snails not in collection, pictures were taken in the field and data could be downloaded from https://observation.org/ (via the app ObsMapp) for occurrences in 2020
2. All data were finally entered together as an occurrence dataset in Microsoft Excel (version 1908) and stored as an Excel Workbook (*.xlsx)
3. Standardisation of dataset to Darwin Core (Wieczorek et al. 2012) (see further) Using R (R Core Team 2019) in RStudio (RStudio Team 2019)Taxonomic information was added based on the scientific name and expressed in kingdom, taxonRank, and nomenclaturalCodeDataset metadata information was added (language, datasetID, institutionCode, datasetName) as well as a unique taxon ID (taxonID), the license, and the rights holder (rightsHolder)The final occurrence dataset was exported as a CSV-file (*.csv)
4. Data publication Using the GBIF Integrated Publishing Toolkit (Robertson et al. 2014) instance at the Belgian Biodiversity Platform (https://ipt.biodiversity.be) (see further)Complete metadataUpload source data (occurrence.csv)Publish on GBIF (https://www.gbif.org/)

## Dataset

### Dataset description

The following Darwin Core terms (https://dwc.tdwg.org/terms/) are used in the dataset: occurrenceID, family, scientificName, identificationQualifier, genus, scientificNameAuthorship, eventDate, year, basisOfRecord, lifeStage, organismQuantity, organismQuantityType, decimalLatitude, decimalLongitude, geodeticDatum, coordinateUncertaintyInMeters, locality, municipality, stateProvince, island, islandGroup, country, countryCode, recordedBy, identifiedBy, identificationRemarks, occurrenceRemarks, associatedMedia, kingdom, taxonID, language, license, rightsHolder, datasetID, institutionCode, datasetName, taxonRank, nomenclaturalCode

**Object name**: Land and freshwater molluscs of Gran Canaria (Spain)

**Format name**: Darwin Core Archive format

**Format version**: 1.0

**Character encoding**: UTF-8

**Language**: English

**License**: https://creativecommons.org/publicdomain/zero/1.0/

**Usage norms**: https://www.inbo.be/en/norms-data-use (Desmet et al. 2014)

**Publication date**: 2020-03-12

**Distribution**: https://ipt.biodiversity.be/resource?r=snail-gran-canaria-occurrences

**DOI**: https://doi.org/10.15468/ny1f9n

## Data records

The data are standardized to Darwin Core (Wieczorek et al. 2012) using an R script based on the TrIAS Checklist Recipe (Reyserhove et al. 2018) with R (R Core Team 2019) in RStudio (RStudio Team 2019). The R script used for mapping the data to Darwin Core can be accessed here: https://github.com/BelgianBiodiversityPlatform/landsnails-occurrences. The data are published using the GBIF Integrated Publishing Toolkit (Robertson et al. 2014) instance at the Belgian Biodiversity Platform (https://ipt.biodiversity.be). The data are organized as an occurrence only dataset, with the occurrence core containing 389 records. The Belgian Biodiversity Platform IPT archives the data and thus serves as the data repository. The data and resource metadata are available for download in the downloads section. The versions table lists other versions of the resource that have been made publicly available and allows tracking changes made to the resource over time.

## Additional information

Empty shells were collected for over 75% of the occurrences in the dataset and deposited in the private collection of first author. For 282 of the 389 observations, links to 101 images of living animals, empty shells and habitats can be found in associatedMedia.

The Darwin Core Archive creation and the publication of the data is part of the ‘Integrated Biodiversity research Project’ course organized in the ‘Master of Science in Biology’ program of Ghent University (Belgium).
